# Distinguishing functional exosomes and other extracellular vesicles as a nucleic acid cargo by the anion‐exchange method

**DOI:** 10.1002/jev2.12205

**Published:** 2022-03-14

**Authors:** Naohiro Seo, Junko Nakamura, Tsuguhiro Kaneda, Hiroaki Tateno, Asako Shimoda, Takanori Ichiki, Koichi Furukawa, Jun Hirabayashi, Kazunari Akiyoshi, Hiroshi Shiku

**Affiliations:** ^1^ Department of Personalized Cancer Immunotherapy Mie University Graduate School of Medicine Mie Japan; ^2^ Core Research for Evolutional Science and Technology (CREST) Japan Science and Technology Agency (JST) Tokyo Japan; ^3^ Research Center for Medical Glycoscience National Institute of Advanced Industrial Science and Technology Ibaraki Japan; ^4^ Department of Polymer Chemistry Graduate School of Engineering Katsura Int'tech Center Kyoto University Kyoto Japan; ^5^ Department of Materials Engineering School of Engineering The University of Tokyo Tokyo Japan; ^6^ Department of Biomedical Sciences Chubu University College of Life and Health Sciences Aichi Japan; ^7^ Institute for Glyco‐core Research (iGCORE) Nagoya University Aichi Japan

**Keywords:** anion‐exchange, exosome, extracellular vesicle, large‐preparation, membrane charge, microvesicle, ultrafiltration

## Abstract

The development of a new large‐scale purification protocol is required for research on the reliable bioactivity and drug discovery of extracellular vesicles (EVs). To address this issue, herein, we propose an effective method for preparing high‐performance exosomes (EXOs) by using an anion‐exchange method. Cytotoxic T‐lymphocyte (CTL) EVs from 4 L of culture supernatant through a 220 nm cut‐off filter are divided into two populations at a deproteinization rate of over 99.97%, which are eluted at low (0.15 M–0.3 M) and high (0.3 M–0.5 M) NaCl concentrations (approximately 2 × 10^12^ and 1.5 × 10^12^ particles, respectively) through the anion‐exchange column chromatography. The former are abundant in EXO proteins, including late endosome‐associated proteins and rab‐family and integrin‐family proteins, and functional micro (mi) RNAs, and have bioactivity for preventing tumour metastasis by depleting mesenchymal cell populations in the primary tumour lesions. By contrast, the latter is microvesicle (MV)‐like particles including DNA, core histone and ribosomal proteins, and GC‐rich miRNAs with unknown function, and are easily phagocytosed by mannose receptor^+^ Kupffer cells. Thus, the anion‐exchange method is suitable for the large‐scale separation of bioactive EXOs and MV‐like EVs as a cargo for dangerous nucleic acids at high‐purity.

## 1 INTRODUCTION

A wide variety of cells, including immune cells, release lipid bilayer membrane extracellular vesicles (EVs) such as late endosome‐derived exosomes (EXOs, also called small‐EVs) and plasma membrane‐budding microvesicles (MVs, also called large‐EVs, including apoptotic bodies, oncosomes, and ectosomes; Colombo et al., 2014) with sizes of 50–200 and 100–2000 nm in diameter (EVs with a diameter of approximately 100–200 nm are a mixture of EXOs and MVs), respectively (Gutiérrez‐Vázquez et al., 2013; György et al., 2011). Non‐membranous exomeres with diameters of less than 50 nm have recently been reported (Zhang et al., 2018). EXOs are derived in the endo‐lysosomal system and are generated as intraluminal vesicles (ILVs) in late endosome (multivesicular bodies [MVBs]) through endosomal sorting complexes required for transport (ESCRT) dependent sorting mediated by the most famous exosome marker proteins such as apoptosis‐linked gene 2‐interacting protein X (Alix) and tumour susceptibility gene (Tsg) 101, and are released by fusing MVBs with the plasma membrane (Colombo et al., 2013; Sette et al., 2010). However, through known ESCRT‐dependent and ‐independent pathways on EXO formation and different packaging mechanisms of micro (mi) RNAs and proteins in EVs from tumour cell lines (Palma et al., 2012; Trajkovic et al., 2008; van Niel et al., 2011), EVs released from a single cell line are structurally heterogeneous populations. In EV drug discovery for clinical use, it is crucial to determine the particle type based on its function and separate and purify it as much as possible.

Unlike MVs of plasma membrane origin, it has been predicted that the EXO membrane is concentrated by sphingolipids represented by sphingomyelin and gangliosides, which are known to prevent ILV‐budding in late endosomes by blocking ceramide biosynthesis, and by accumulating tetraspanin molecules such as CD9, CD63, and CD81; glycosyl‐phosphatidylinositol‐anchored proteins such as CD90; G protein‐coupled receptors; and cholesterols (de Gassart et al., 2003; Estelles et al., 2007; Trajkovic et al., 2008; Wubbolts et al., 2003). It was reported in a study using fractionated EVs with a high‐resolution density gradient that EXOs do not contain DNA (Jeppesen et al., 2019). Moreover, a report indicated the DNA‐embedding of large‐EVs, but not small‐EVs, in the plasma of patients with prostate cancer (Vagner et al., 2018). All types of EVs have been widely thought to exhibit a negative charge because anionic phosphatidylserine (PS), localized at the inner leaflet of the plasma membrane of viable cells, is exposed on the outer leaflet of the EV membrane, as shown in plasma membrane of dead cells (Fujii et al., 2015; Matsumoto et al., 2017). Interestingly, some studies have reported that EXOs have a weaker affinity for annexin V and lactadherin (Heijnen et al., 1999; Jeppesen et al., 2019; Matsumura et al., 2019), both PS‐binding proteins, than other EVs, suggesting that EXOs can be separated from plasma membrane‐originated EVs based on the difference in the negative charge of the membrane.

Ultracentrifugation (UC)‐based isolation techniques are widely used as the gold standard method for characterizing the biological significance of EVs (Lobb et al., 2015; Momen‐Heravi et al., 2013). In addition to being unable to distinguish among MVs, EXOs, and exomeres, insoluble aggregated proteins in the culture supernatant are also precipitated together with EVs when applying the UC method (Baranyai et al., 2015; Willms et al., 2016). Aggregation between EVs and the nonspecific binding of culture medium‐derived proteins with EVs significantly impair the reliability of the biological activity of UC EVs under the difficulties inherent to quantitative experiments (Linares et al., 2015; Xu et al., 2016). Density gradient centrifugation using iodixanol and affinity separation using mAbs specific for CD9, CD63, and CD81 tetraspanin molecules, and T‐cell immunoglobulin and mucin domain‐containing molecule‐4 (TIM‐4) specific for PS are frequently utilized in EV preparation, although UC‐concentrated EVs are utilized under such settings (Nakai et al., 2016; Stranska et al., 2018; Zhang et al., 2018). Although density gradient and affinity‐based methods can obtain EVs at high purity, it is difficult to prepare a large number of EVs for physicochemical analysis and investigating the detailed biological properties of EVs. In addition, EVs with low negative membrane charges are likely to be missed during the PS‐targeting preparation method.

We have clarified in the murine study that CD8^+^ CTL EVs prevent tumour metastasis by modulating mesenchymal cell populations in the primary tumor lesions via their content, such as miR‐298‐5p (Seo et al., 2018). In this study, we describe a large‐scale EV preparation method at high‐purity based on this biological property using an anion‐exchange method, and clarify the detailed physiological characteristics, including protein, membrane lipid, and miRNA distributions, and DNA content, surface glycosylation, and target cell specificity. We conclude that the anion‐exchange method is the most suitable for a good manufacturing practice (GMP)‐compliant EV preparation in clinical application.

## 2 MATERIALS AND METHODS

### 2.1 Mice

Female BALB/c mice were purchased from Japan SLC. H‐2K^d^‐restricted and mutated (m) ERK2 136–144 peptide (CTL epitope of CMS5a and CMS5m fibrosarcomas)‐specific TCR‐transgenic DUC18 mice (Hanson et al., 2000; Ikeda et al., 1997), I‐A^b^‐restricted OVA 323–339 peptide‐specific T cell receptor (TCR)‐transgenic OT‐II mice, and BALB/c mice were maintained at the Experimental Animal Facility of Mie University, and used at 8–10 weeks of age. The Ethics Review Committee for Animal Experimentation of Mie University approved the experimental protocols (approval no.: 23–8).

### 2.2 Cells

Human peripheral blood mononuclear cells (PBMCs) were purchased from Takara Bio Inc. NK‐92, HEK293.2sus, and Jurkat E6‐1 cells were purchased from the American Type Culture Collection. The experimental procedures of human study were approved by the ethics review committee of the Mie University Graduate School of Medicine (approval no.: 2879). Bone‐marrow‐derived MSCs from BALB/c were purchased from Cyagen Biosciences, and cultured in the MesenCult Expansion Kit for mouse (Veritas). CMS5a and CMS5m cells were cultured in an RPMI‐1640 medium supplemented with 10% fetal calf serum (FCS). KUP5 cells (immortalized Kupffer cell [C57BL/6 origin] line) were provided by Dr. H. Kitani (National Agriculture and Food Research Organization; Kitani et al., 2014), and cultured in Dulbecco's‐modified Eagle's Medium (DMEM) (high glucose) supplemented with 10% FCS, 10 μg/ml bovine insulin (Sigma–Aldrich), and 250 μM monothioglycerol (Fujifilm).

### 2.3 Preparation of culture supernatants

EV‐depleted FCS (dFCS) was prepared by ultracentrifugation at 150,000 × *g* for 18 h. Murine CTLs (CD8^+^ T cells) and T helper cells (CD4^+^ T cells) were obtained from the splenocytes of DUC18 and OT‐II mice, respectively. Splenocytes were cultured for three days at a concentration of 1 × 10^6^ cells/ml in an RPMI‐1640 medium containing 1 μg/ml of peptide (mERK2 136–144, QYIHSANVL for CTL; OVA 323–339, ISQAVHAAHAEINEAGR for Th cells) and 10% dFCS, and then supplemented with 100 international (I) U/ml recombinant (r) IL‐2 (Novartis) for an additional four days (for a total of seven days). Human PBMCs (0.5 × 10^6^ cells/ml) were cultured in 2 μg/ml anti‐CD3 mAb (Biolegend, OKT3)‐immobilized six‐well plates in an RPMI‐1640 medium supplemented with 10% dFCS, 1 μg/ml anti‐CD28 mAb (Biolegend: CD28.2), and 200 IU/ml rIL‐2 (Novartis) for three days, and then cultured for an additional seven days by adding the same volume of fresh RPMI‐1640 medium without anti‐CD28 mAb every other day. NK‐92 cells were cultured in an RPMI‐1640 medium supplemented with 10% dFCS and 200 IU/ml rIL‐2 (Novartis) at 2 × 10^5^ cells/ml for seven days. Jurkat 6E‐1 cells were cultured in an RPMI‐1640 medium supplemented with 10% dFCS at 2 × 10^5^ cells/ml for seven days. Nonadherent HEK293.2sus cells were cultured in a 293 SFM II medium (Thermo Fisher Scientific) at 5 × 10^5^ cells/ml in 100‐ml Erlenmeyer flasks (Corning) under a 100‐rpm rotation and 8% CO_2_ concentration. The culture supernatants were obtained first centrifuged at 10,000 × *g* for 20 min to deplete the cells and passed through 0.45‐ and 0.22‐μm filters to remove cell debris and aggregated proteins.

### 2.4 EV preparation using ultracentrifugation (UC)

The culture supernatants were subjected to UC at 120,000 × *g* (SW28 rotor, Beckman Coulter) for 2 h. The obtained EV‐containing pellets were suspended in PBS, recentrifuged at 120,000 × *g* for 2h, dissolved in 0.5–2 ml PBS, and stored at 4°C.

### 2.5 Concentration and deproteinization of culture supernatants by ultrafiltration (UF)

UF of the culture supernatant was performed using a tangential flow filtration (TFF) system (KrosFlo Research IIi TFF system, Silicon tube #16 [ACTUE1625N], Spectrum) using mPES MidiKros Filter Modules (D02‐E750‐05‐N; MWCO 750 kDa, Spectrum) at an entrance flow rate of 50 ml/min. Culture supernatants were concentrated by over 20‐hold, and substituted with more than 400 ml PBS (approximately 500–2000 ml of culture supernatant) or 1 L PBS (4‐L culture supernatant). The obtained concentrates were subjected to an anion‐exchange column chromatography after measuring the particle number, particle diameter, and protein concentration.

### 2.6 Anion‐exchange column chromatography

Anion‐exchange column chromatography was performed using DEAE‐Sepharose Fast Flow (GE Healthcare). A DEAE‐sepharose column (10 ml for EVs from 500‐ml culture supernatant, and 50 ml for EVs from 4‐L culture supernatant) was equilibrated with 10 mM Tris‐HCl (pH7.4) containing 0.15 M NaCl (TBS). The EV‐containing PBS solutions concentrated with MWCO 750 kDa UC was loaded into the column and washed with TBS (over five column volumes). EVs bound with DEAE‐sepharose were eluted using a NaCl linear gradient (0.15 M to 0.8 M NaCl) or stepwise method (0.3 M and 0.5 M NaCl), and fractionated at each 3.5 ml. The electrical conductivity of each fraction was then measured using LAQUAtwin (Horiba Scientific) to calculate the exact NaCl concentration, and immediately returned to the saline concentration (0.15 M) with 10 mM Tris‐HCl (pH7.4).

### 2.7 Observations of mesenchymal cell population and lung metastasis after treatment of EVs to subcutaneous tumours

**FIGURE 1:**
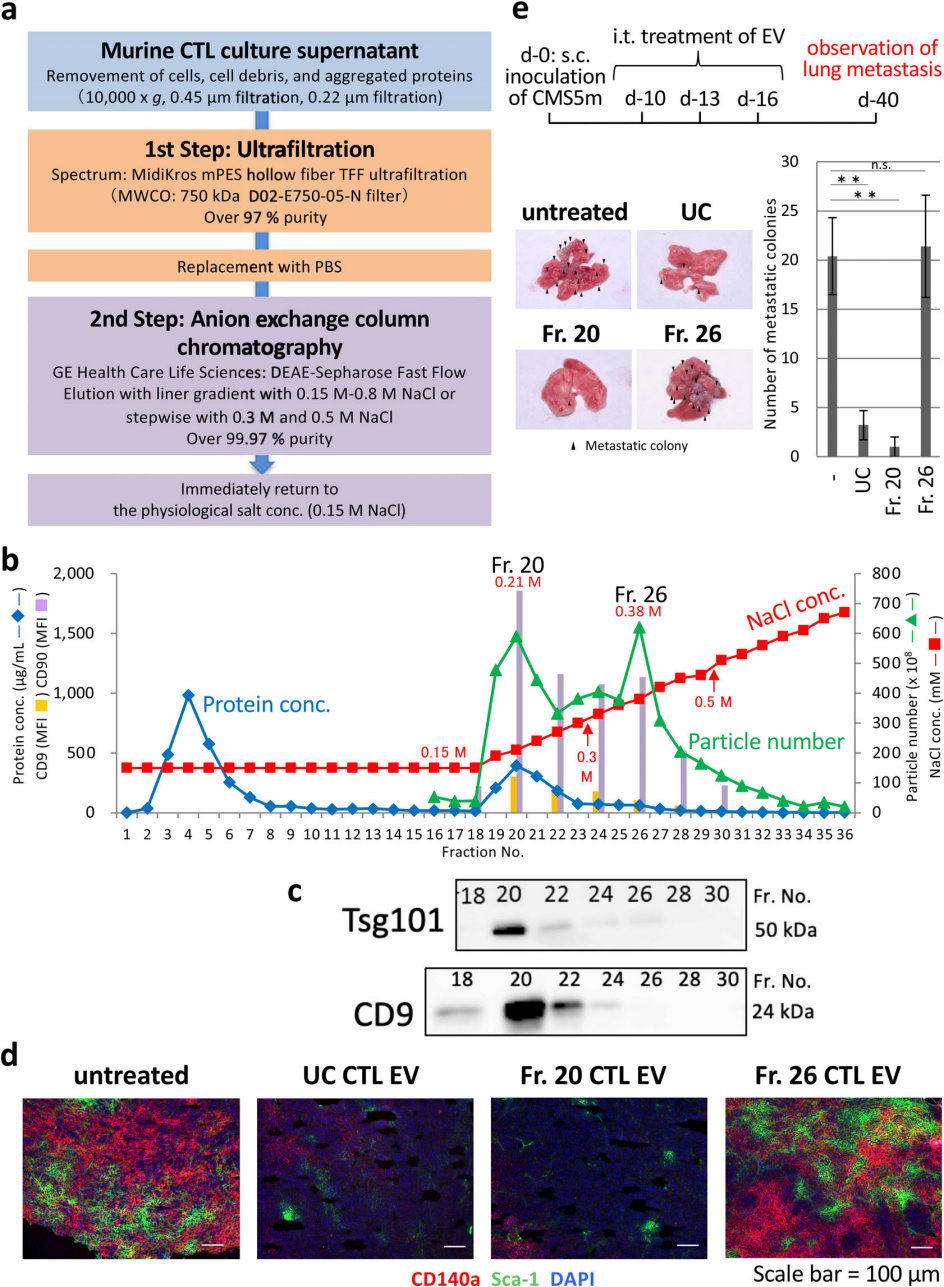
Elution of bioactive CTL EVs at low‐salt concentrations by DEAE column chromatography. (a) Preparation workflow of CTL EVs by DEAE column chromatography after concentration and deproteinization of culture supernatant with 750 kDa MWCO UF. (b) A typical elution pattern by NaCl linear gradient (0.15 M–0.8 M) of CTL EVs from DEAE column chromatography based on particle number, protein concentration, and expressions of CD9 and CD90 (FACS analysis). (c) Western blot analysis of CTL EVs of the indicated fractions by CD9 and Tsg101 mAbs. (d) Fr. 20 and Fr. 26 CTL EVs, and UC CTL EVs (2 × 10^9^ EVs/tumour) were treated i.t. in subcutaneous CMS5a tumours (fibrosarcoma: 1 cm in diameter). On day 3 after treatment, tumours were resected, and cryo‐sections of tumours were stained with PE‐CD140a mAb, FITC‐Sca‐1 mAb, and DAPI. The photograph is representative of three photographs. (e) *Upper*: Protocol for the study of lung tumour metastasis using fractionated CTL EVs. Fr. 20 and Fr. 26 CTL EVs (2 × 10^9^ EVs/tumour) were injected i.t. on three consecutive days in subcutaneous CMS5m tumours (metastatic fibrosarcoma). On day 40, the lungs were removed, and metastasis colonies were observed. The photograph is representative of three photographs of the lungs from three mouse (*lower left*). The number of metastatic colonies obtained from the 20‐ and UC‐treated groups were significantly fewer than those from untreated‐ or Fr.26‐treated group (*n* = 3; the data express the mean ± SD; **p < 0.01, n.s., not significant, Student's *t‐*test) (*lower right*)

CNS5a (2 × 10^5^ cells) were inoculated subcutaneously into the back skin of BALB/c mice. On day 12, 2 × 10^9^ EVs (Fr. 20, 26 [See Figure 1b], and UC) were injected intratumorally. Three days after EV treatment, the CMS5a tumour was resected and subjected to immunohistochemistry using frozen sections. Frozen CMS5a specimens embedded in an O.C.T compound (Sakura Finetech) were sectioned at a thickness of 3 μm, air‐dried for 2 h, and fixed with ice‐cold acetone (Fujifilm) for 15 min. After washing three times with PBS, the tissue slides were incubated at 4°C in a blocking solution (PBS supplemented with 1% BSA, 5% Blocking One Histo [Nacalai Tesque], and 0.2 μg/ml anti‐mouse CD16/CD32 mAb [93, Biolegend]) for 30 min. The tumour sections on the slides were then labelled with phycoerythrin (PE)‐conjugated anti‐mouse CD140a mAb (APA5, Biolegend) and fluoresceine isothiocyanate (FITC)‐conjugated anti‐mouse Sca‐1 mAbs (D7, Biolegend) diluted with PBS supplemented with 1% BSA and 5% Blocking One Histo for 1 h at room temperature in a humidified chamber. After washing three times with PBS supplemented with 0.02% Tween‐20, the slides were mounted in a Prolong Gold antifade reagent with DAPI (Life Technologies), and observed with fluorescence microscopy (BX53F; Olympus Co., Ltd.). The photographs from PE‐, FITC‐, and DAPI‐stained tissue sections were merged using ImageJ for Mac OS X software (Adobe Systems Software). Metastatic CMS5m cells (2 × 10^5^ cells) were inoculated subcutaneously into the back skin of BALB/c mice. On days 10, 13, and 16, 2 × 10^9^ particles of EVs (Fr. 20 and 26 [see Figure 1b], and UC) were injected intratumorally, and metastatic lung colonies were observed on day 40 (*n* = 3).

### 2.8 Characterization of EVs

The protein concentration of EVs was assessed using a bicinchoninic acid (BCA) protein assay kit (Pierce) according to the manufacturer's instructions. The mean number and diameter of EVs were measured using a nano‐tracking analysis (NTA; LM10‐HS, NanoSight). EV surface proteins were detected using a flow cytometric analysis (BD, FACS Canto II) of latex beads (4 μm in diameter, Thermo Fisher Scientific) bound EVs according to the manufacturer's instructions. Latex beads were mixed with EVs at a ratio of three particles/bead in 0.1 M 2‐morpholinoethanesulfonic acid (MES) buffer, incubated for 2 h on a rotating shaker, and then blocked with 400 mM glycine. EVs‐bound beads were washed twice with PBS containing 2% dFCS, stained with PE‐conjugated anti‐mouse CD9 mAb (MZ3, Biolegend), anti‐mouse CD90 mAb (53‐2.1, Biolegend), anti‐mouse MHC‐II (I‐A/I‐E) mAb (M5/114.15.2, Biolegend) and anti‐human CD81 mAb (5A6, Biolegend), or fluorescein‐conjugated LEL (Vector Lab), UEA‐I (Vector Lab), and recombinant (r) LSLN (Fujifilm) for 30 min at 4°C, washed three times with PBS, and subjected to a flow cytometric analysis (BD, FACS Canto). EV DNA was extracted using an Exosomal DNA Extraction kit (101 Bio) according to the manufacturer's instructions. EVs (2 × 10^9^ particles/ml) were treated with or without 50 unit/100 μl DNase I (Thermo Fisher Scientific). After 15 min of incubation at room temperature, DNase I was inactivated with 20 μl of 50 mM EDTA per 100 μl. DNase I‐treated EVs or untreated EVs were subjected to an Exosomal DNA Extraction kit. The absorbance (260 nm) of the extracted DNA was analyzed using a NanoDrop 1000 spectrophotometer (Thermo Fisher Scientific). Recombinant LSLN lectin was prepared using an *Escherichia coli* expressing vector according to a previous method (Tateno et al., 2011). Fluorescein‐conjugated LEL, UEA‐I, and rLSLN were obtained using the Fluorescein Labeling kit (Dojindo) according to the manufacturer's instructions.

### 2.9 Detection of EV uptake by mesenchymal stem cells (MSCs) and Kupffer cells

The engulfment of EVs by MSCs and KUP5 was examined using SYTO RNASelect (Thermo Fisher Scientific)‐stained EVs. EVs (5 × 10^9^ particles/200 μl PBS) were labelled with 10 μM SYTO RNASelect green fluorescence at 37°C for 20 min, and then passed through a PD SpinTrap™ G‐25 (GE Healthcare) spin column to exclude free dye. To estimate the quantity of total RNAs, SYTO RNASelect‐stained EVs were mixed with latex beads (4 μm in diameter, Thermo Fisher Scientific) at a ratio of three particles/bead in a 0.1 M MES buffer, incubated for 2 h on a rotating shaker, and then treated with 400 mM glycine. SYTO RNASelect‐stained EVs bound with beads were washed twice with PBS containing 2% dFCS, and then subjected to a flow cytometric analysis (BD, FACS Canto). SYTO RNASelect‐stained EVs were added to the culture of MSCs or Kupffer cells at an EV concentration of 2 × 10^9^ particles/ml, and incubated for 2 h (for Kupffer cells), 4 h (for MSCs), and 24 h (for both cells). After washing with PBS, cells obtained were fixed with methanol at room temperature for 20 min, dried at room temperature for 1 h, and embedded with a Prolong Gold Antifade Reagent with DAPI (Thermo Fisher Scientific). EV‐treated MSCs and Kupffer cells were observed using fluorescence microscopy (BX53 with DP73 camera, Olympus) and flow cytometry (BD, FACS Canto II). The expressions of mesenchymal stem cell markers, M2 macrophage markers, and fucose/mannose receptors on MSCs and Kupffer cells were examined by a flow cytometry (BD, FACS Canto II) after staining (room temperature, 30 min) with PE‐conjugated anti‐mouse mAb to CD45 (30‐F11), CD11b (M1/70), F4/80 (BM8), CD140a (APA5), Sca‐1 (D7), CD206 (C068C2), CD207 (4C7), or CD209a mAbs (MMD3) (all from Biolegend).

### 2.10 Western blot analysis

EVs were dissolved in a Laemmli sample buffer (Bio‐Rad) with or without 5% 2‐mercaptoethanol (ME) (Bio‐Rad), and boiled for 5 min. Proteins from 1 × 10^9^ EVs and 5 μg molecular weight markers (MagicMark XP Standard, Thermo Fisher Scientific) were loaded onto 10% polyacrylamide gel electrophoresis (e‐PAGEL E‐R10L: ATTO) using a sodium dodecyl sulfate (SDS) running buffer (Bio‐Rad) at 40 mA for 60 min. The gel obtained was soaked three times in a transfer buffer (Bio‐Rad), and proteins were transferred onto an Immobilon‐P polyvinylidene fluoride membrane (Merck) with a Trans‐Blot SD Semi‐Dry Transfer Cell (Bio‐Rad) at 1 mA/cm^2^ for 1 h, blocked with 5% skim milk (Fujifilm) in 0.05% TTBS (TBS containing 0.05% Tween 20), and then incubated overnight with a primary Ab to mouse Tsg101 (EPR7130(B), Abcam), CD9 (KMC8, Thermo Fisher Scientific), CD90 (778053, R&D systems), Flotillin‐2 (C42A3, Cell Signalling Technology), integrin αV (D2N5H, Cell Signalling Technology), β‐actin (2F1‐1, Biolegend), annexin A1 (BL28553, Biolegend), annexin A2 (D11G2, Cell Signaling Technology), or annexin A6 (rabbit polyclonal, Proteintech) at 4°C. After washing three times with 0.05% TTBS, the membrane was incubated with horseradish peroxidase‐conjugated secondary Ab to mouse IgG (GE Healthcare), rat IgG (R&D Systems), or rabbit IgG (MBL Life Science) at room temperature for 1 h. After washing four times with 0.05% TTBS, the membranes were treated with ECL prime (GE Healthcare) and visualized using LAS‐4000 (Fujifilm).

### 2.11 Determination of accurate EV concentration by NTA

The range of accurate particle concentrations by NTA (NanoSight: LM10‐HS) was examined through a serial dilution of UC CTL EVs or weak negatively charged polystyrene beads (100 nm in diameter, 4.55 × 10^13^/ml, Polysciences Inc.). As indicated in Figure S1, the range of 1 × 10^8^–1 × 10^9^ particles/ml under a constant sensitivity (camera level 12) showed almost accurate particle concentrations. The exact particle number of EVs in all experiments were calculated by measurement in this range and used it for comparison.

### 2.12 Cryo‐transmission electron microscopy (TEM)

Cryo‐TEM was conducted at the Nara Institute of Science and Technology (NAIST), supported by the Nanotechnology Platform Program (Synthesis of Molecules and Materials, S‐19‐NR‐0013) of the Ministry of Education, Culture, Sports, Science and Technology (MEXT), Japan. Briefly, using a micropipette, 1 μl of 5 × 10^9^ particles/ml of L‐s, H‐s, and UC CTL EVs was dropped on a Cu microgrid (JEOL Ltd., 1643) that had been subjected to a hydrophilic treatment, and set in the cryo‐sample preparation device (Laica Microsystems, EM‐CPC). Excess solution was wiped off with filter paper, and the grid was immediately placed in liquid ethane. The samples were quickly frozen and subjected to 300 kV‐TEM (JEOL Ltd., JEM‐3100FEF).

### 2.13 Measurement of zeta potential values of EVs

It is impossible to measure the zeta potential in physiological NaCl (0.15 M) solutions, such as PBS or TBS, because the ionic strength is too high. We conducted a preliminary study on the stability of EVs in water (MilliQ: Merck). As indicated in Figure S4, it was found that 80% of CTL EVs (1 × 10^9^ particles/ml) from ultrafiltration maintained a stable dispersion and retained the spherical shape despite the doubling in volume (20 % increase in diameter) at 4°C after 24 h. Therefore, we decided to measure the zeta potential value of EVs in 10 mM Tris‐HCl (pH 7.5) containing 0.015 M NaCl (one‐tenth of the physiological salt concentration). The zeta potential values were measured using a Nanopartica SZ‐100 (Horiba) with a glass cuvette (Horiba) at an EV concentration of 1 × 10^9^ particles/ml.

### 2.14 Microarray analysis of miRNAs

Microarray of EV miRNAs was conducted in Toray Industries using 3D‐Gene device. Briefly, total RNAs were extracted from 1 × 10^11^ particles of L‐s and H‐s CTL EVs using TRIzol (Thermo Fisher Scientific) according to the manufacturer's instructions. The total RNAs were labelled using a 3D‐Gene miRNA labelling kit (Toray Industries). Labelled RNAs were hybridized onto a 3D‐Gene Mouse miRNA Oligo chip (Toray Industries). The annotation and oligonucleotide sequences of the probes conformed to miRBase (http://microrna.sanger.ac.uk/sequences/). After stringent washes, fluorescent signals were scanned using a 3D‐Gene Scanner (Toray Industries) and analyzed using 3D‐Gene Extraction software (Toray Industries). The relative expression level of the given miRNAs was calculated by comparing the signal intensities of the valid spots throughout the microarray experiments. The data were globally normalized per array, such that the median signal intensity was adjusted to 25.

### 2.15 Proteome analysis

An exhaustive proteomic analysis of L‐s and H‐s CTL EVs was conducted using quantitative LC‐MS/MS of iTRAQ‐ labelled EV peptides (Intégrale Co., Ltd.). Briefly, L‐s and H‐s CTL EVs were digested with trypsin (AB Sciex) and labelled with iTRAQ (iTRAQ Reagent‐8 Plex Kit, AB Sciex). Reporter tags 114 and 115 were used for the isolated peptides of L‐s and H‐s CTL EVs, respectively. The labeled samples were combined and fractionated into six fractions through strong cation exchange chromatography and desalted using a MonoSpin® C18 column (GL Science). Each peptide fraction was analyzed using Q Exactive Plus (Thermo Fisher Scientific) coupled on‐line with a capillary HPLC system (EASY‐nLC 1200, Thermo Fisher Scientific) to acquire MS/MS spectra. Proteome Discoverer (version 2.1, Thermo Fisher Scientific) was used to search against the protein database SWISS‐Prot. iTRAQ label‐based quantification was applied.

### 2.16 Lipidomics

Lipid analysis was conducted in Kazusa DNA Research Institute by unbiased lipidomics applied previously (Tsugawa et al., 2017). Briefly, the dried EV fraction was dissolved in 320 μL of chloroform/methanol/water (1/2/0.2) and sonicated for 2 min. After 40 min of incubation at 20°C, the mixture was centrifuged at 1670 × *g* for 10 min at 20°C, and the supernatant was transferred to an LC vial. An LC‐MS/MS analysis was carried out using a quadrupole time‐of‐flight (Q‐TOF)/MS (TripleTOF 6600; Sciex Co. Ltd.) coupled with an ACQUITY UPLC system (Waters Co. Ltd,). LC separation was applied with a gradient elution of mobile phase A (methanol/acetonitrile/water [1/1/3, v/v/v] containing 5 mM ammonium acetate [Fujifilm Wako Chemicals], 10 nM EDTA [Dojindo]) and mobile phase B (isopropanol containing 5 mM ammonium acetate and 10 nM EDTA). The flow rate was 300 μl/min at 45°C, and the injection volume was 1 μl. The information‐dependent acquisition (IDA) mode was used to confirm each of the lipid structures in negative‐ion mode. MS‐DIAL and MS‐FINDER software were used for the extraction of sphingolipids and phospholipids, and quantified using multiQuant software.

### 2.17 Lectin microarray analysis

Lectin microarray analysis was conducted according to a previously reported method (Tateno et al., 2011). After adjusting the EV number to 2 × 10^9^ particles/ml with PBST (10 mM PBS, pH 7.4, 140 mM NaCl, 2.7 mM KCl, 1% Triton X‐100), the hydrophobic fraction was labelled with Cy3 NHS ester (GE Healthcare), and excess Cy3 was removed using Sephadex G‐25 columns (GE Healthcare). After dilution with a probing buffer at 0.5 μg/ml, the Cy3‐labeled hydrophobic fraction was applied to the lectin microarray (Rexxam(96) array: GlycoTechinica) and incubated at 20°C overnight. After washing with a probing buffer, fluorescence images were acquired using an evanescent field‐activated fluorescence scanner (GlycoStation™ reader 1200, GP BioSciences). The fluorescence signal of each spot was quantified using Array Pro Analyser (version 4.5, Media Cybernetics), and the background value was subtracted. The background value was obtained from the area without lectin immobilization. The lectin signals of triplicate spots were averaged and normalized to the mean value of 96 lectins immobilized on the array.

### 2.18 Statistical analysis

Student's *t*‐test and Fisher's extract test were used in this study. Statistical significance was set at *p* < 0.05 or *p *< 0.01.

### 2.19 Data availability

Proteomic and lipidomic data were deposited in the PRIDE under accession numbers PXD017850 and PXD017899, respectively. Data of miRNA and lectin microarrays have been deposited in the GEO under accession numbers GSE146817 and GSE146818, respectively. All other data are available from the corresponding author upon request.

## 3 RESULTS

### 3.1 Elution of the functional EV using low salt in anion‐exchange method

First of all, we measured the range showing the exact number of particles through a nano‐tracking analysis (NTA: LM10‐HS [NanoSight]) using murine UF CTL EVs and polystyrene beads, and determined the appropriate range (1 × 10^8^ to 1 × 10^9^ particles/ml at camera level 12) to evaluate EVs in this study (Figures S1a,b).

Figure 1a shows the protocol for EV preparation in this study. EXOs and MVs are the lipid bilayer spherical vesicles with diameters of approximately 50–2000 nm. Ultrafiltration (UF) with a 750 kDa MWCO filter with a diameter of approximately 50 nm may be great useful for the concentrations of EXOs and MVs in the culture supernatant, excluding contaminants such as unnecessary aggregated proteins and exomeres of less than 50 nm in diameter. As expected, the culture supernatant from murine CTLs through a 220 nm cut‐off filter was effectively concentrated over 20‐hold with almost no leakage of particles at over 97% purity by using 750 kDa MWCO UF (Table S1 and Figure S2a).

Anion‐exchange column chromatography (DEAE sepharose) was applied to obtain a high purity fraction of murine CTL EVs from the UF concentrate. In a preliminary study, it was confirmed that HEK293.2sus cells secrete EVs at high numbers (1 × 10^10^ particles/ml in a medium), and approximately 3–5 × 10^11^ particles of HEK293.2sus EVs are retained in an 8‐ml column volume. It has been revealed that murine CTLs maximally produce EVs at 5 × 10^11^ particles per 500 ml of the culture supernatant. Therefore, 5 × 10^11^ particles of UF‐concentrated CTL EVs were subjected to a DEAE column chromatography in a 10‐mL column volume. As shown in Figure 1b, CTL EVs were fractionated by eluting at a linear gradient from saline (0.15 M) to 0.8 M NaCl concentrations into two large peaks (Fractions 20 and 26). The recovery rate determined from the total CTL EV number of all fractions by DEAE column chromatography from UF concentrate was 66.4%, which was higher than the recovery rate of 45.0% in the case of UC. Flow cytometric analysis and western blotting showed the expression of CD9 and Tsg101 in the fractionated EVs of the first peak eluted at low NaCl concentrations in contrast to the broad expression of CD90 in all EV fractions (Figure 1c), suggesting that EXO eluates under low salt concentrations using the anion‐exchange method. Similar results were observed with a DEAE column of separated mouse ovalbumin‐specific Th cell (OT‐II) EVs, human natural killer cell (NK‐92) EVs, and human PBMC EVs (Figure S2b).

CTL EVs in 20 and 26 fractions were examined the effects on the tumour mesenchymal cells, and tumour invasion and metastasis (Seo et al., 2018). Intratumoral (i.t.) administration of fraction 20 CTL EVs showed a strong elimination activity of mesenchymal cell populations (CD140a^+^and/or Sca‐1^+^) compared with no efficacy in a 26 fraction CTL EV‐treated case (Figure 1d). Moreover, lung metastasis of subcutaneous metastatic sarcoma was prevented by i.t. treatment of fraction 20 CTL EVs, but not fraction 26 CTL EVs, indicating the elution of functional CTL EVs at low NaCl concentrations in DEAE column chromatography (Figure 1e). Thus, we were able to separate bioactive CTL EVs and other CTL EVs from culture supernatant.

### 3.2 CTL EVs eluted under low and high salt concentrations have different particle morphology and size

**FIGURE 2:**
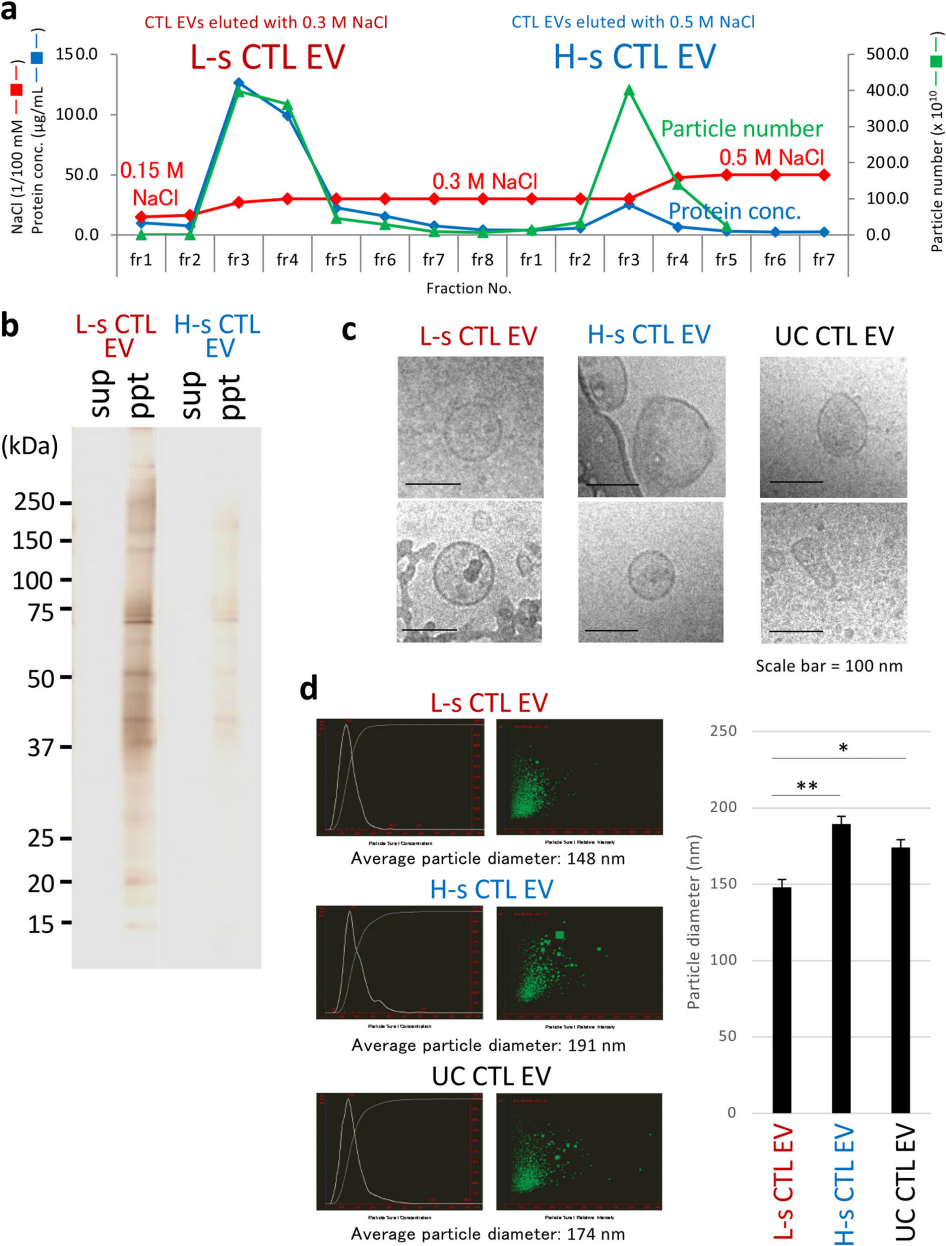
Differences in Morphology and Particle Size between the Purified L‐s and H‐s CTL EVs. (a) Using a stepwise elution method with 0.3 M NaCl and 0.5 M NaCl of DEAE column chromatography (See Figure 1b for the positions of 0.3 M NaCl and 0.5 M NaCl in the NaCl linear gradient), CTL EVs were separated into two populations termed as low‐salt (L‐s) and high‐salt (H‐s) CTL EVs. (b) L‐s and H‐s CTL EV fractions were ultracentrifuged (150,000 × *g*, 2 h), and supernatants (sup) and pellets (ppt) were subjected to silver staining after SDS‐PAGE. (c) L‐s, H‐s, and UC CTL EVs were observed through cryo‐TEM. Two photographs are representative of the four photographs. (d) Particle sizes of L‐s, H‐s, and UC CTL EVs were examined using a nano‐tracking analysis. The particle size was measured 24 times in each L‐s, H‐s, or UC group. *Left*: The data are typical nano‐tracking analysis images. *Right*: Particle sizes obtained from L‐s CTL EVs were significantly smaller than those from H‐s or UC CTL EVs (The data express the mean ± SD; **p < 0.01, *p < 0.05, Student's *t‐*test)

To examine the particle characteristics of CTL EVs eluted at low and high NaCl concentrations, UF‐concentrated CTL EVs (4 × 10^12^ particles from 4 L culture supernatant) were subjected to 50‐mL DEAE column chromatography and eluted at 0.3 M (L‐s, low‐salt: approximately 2 × 10^12^ particles) and 0.5 M (H‐s, high‐salt: approximately 1.5 × 10^12^ particles) NaCl concentrations using a stepwise method (Figure 2a). Because the total amount of proteins eluted through an anion‐exchange method was below 0.03% of the total proteins in the culture supernatant, the deproteinization rate of CTL EVs was over 99.97%. No proteins were detected by silver staining after SDS‐polyacrylamide gel electrophoresis of the supernatant of the L‐s and H‐s CTL EV fractions, indicating that almost all of the 0.03% proteins eluted through an DEAE column are EV‐associated proteins (Figure 2b). A morphological observation using cryo‐TEM showed that L‐s CTL EVs, H‐s CTL EVs, and UC CTL EVs were respective spherically sharp with the same size, unequal sizes including a remarkably large size, and many sharp distortions with a lipid bilayer membrane (Figure 2c). In addition, NTA revealed that L‐s CTL EVs have smaller particle size than H‐s CTL EVs or UC CTL EVs (Figure 2d).

### 3.3 L‐s and H‐s CTL EVs exhibit the features of EXO and MV‐like DNA cargo, respectively

**FIGURE 3:**
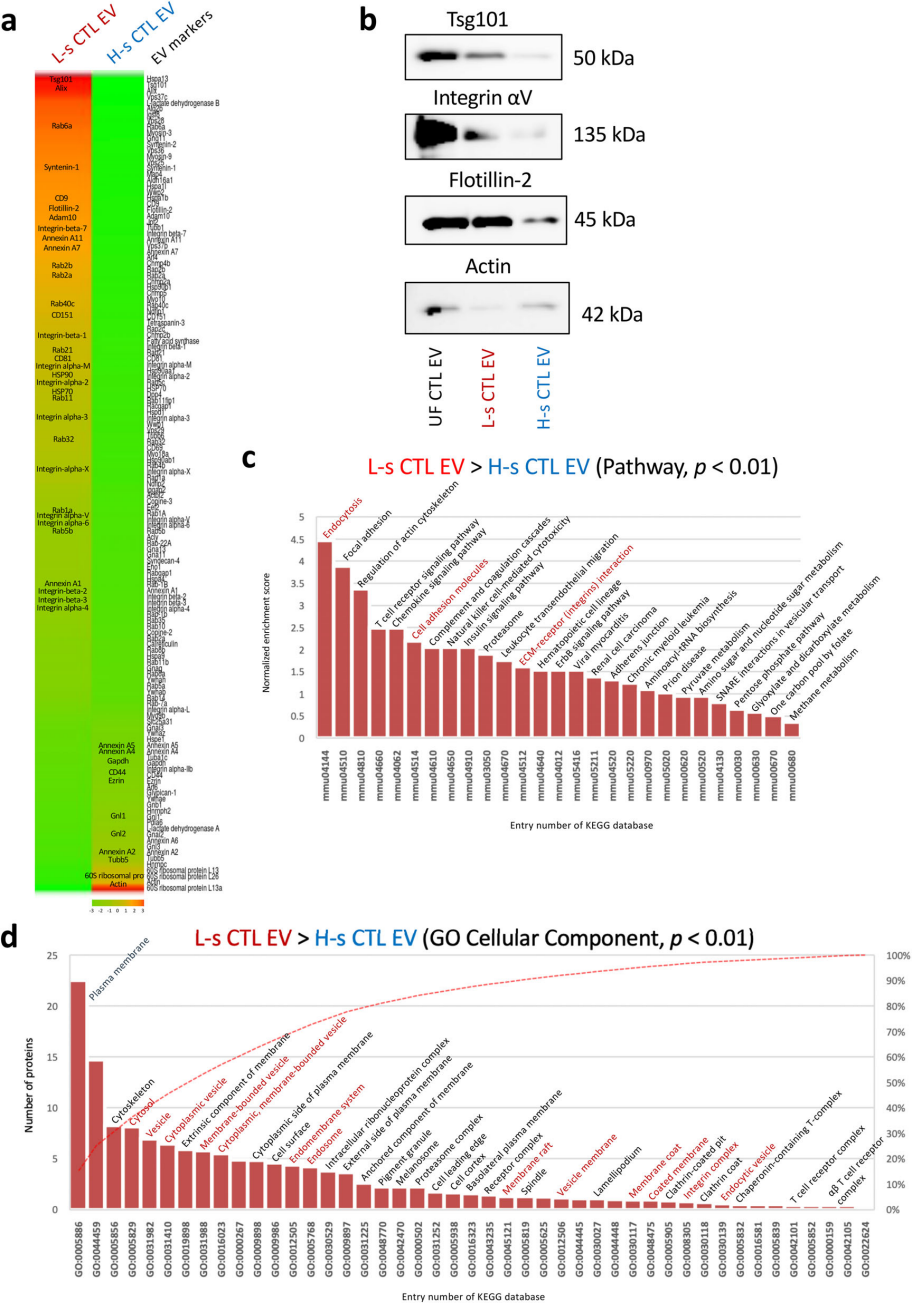
L‐s CTL EVs as the exosomes of late endosome origin. (a) Abundance of L‐s CTL EV and H‐s CTL EV proteins is showed by a heat map of reported EV marker proteins. Crucial EV proteins are described in the heat map column. (b) To confirm the abundance ratio, a western blot analysis of L‐s, H‐s, and UC CTL EV proteins was conducted using Tsg101, Integrin αV, Flotillin‐2, and Actin mAbs. (c) A pathway analysis was conducted for all L‐s CTL EV‐dominant proteins (*p* < 0.01, Fisher's extract test) compared with H‐s CTL EV proteins using Kyoto Encyclopedia of Genes and Genomes (KEGG) database. (d) L‐s CTL EV‐dominant proteins (*p* < 0.01, Fisher's extract test), compared with H‐s CTL EV proteins were analyzed using the gene ontology (GO) cellular component (KEGG database). Analyzes of the pathway and GO cellular component were applied using DAVID Bioinformatics Resources 6.8. Statistical significance was set at *p* < 0.05, and only *p *< 0.01 was shown.

A proteome analysis using iTRAQ revealed that L‐s CTL EVs had EXO markers, including heat shock protein (HSP) 70, alix, HSP90, Tsg101, flotillin‐2, CD9, CD81, rab‐family proteins (Rab6a, 2b, 2a, 40c, 21, 11, 32, 1, and 5b), annexin‐family proteins (Annexin‐A11, ‐A7, and ‐A1), and integrin‐family proteins (Integrin‐β7, ‐β1, ‐αΜ, ‐α2, ‐α3, ‐αX, ‐αV, ‐α6, ‐β2, ‐β3, and ‐α4), compared with H‐s CTL EVs. By contrast, H‐s CTL EVs were abundant in cytoskeleton molecules (actin and ezrin), housekeeping molecule (GAPDH), ribosomal proteins, annexin A2, major histocompatibility complex H‐2 class II molecules, and ADP‐ribosylation factor 6 (ARF6) (Figures 3a,b, S3a,b,c). A pathway analysis of L‐s CTL EV‐dominant proteins (*p* < 0.01) detected endocytosis mainly consisting of clathrin‐dependent endocytosis‐related proteins and late endosome‐related proteins as the top, demonstrating that L‐s CTL EVs are classified as EXOs (Figures 3c,d, S4a). In addition, integrins for interacting extracellular matrix (ECM) proteins exhibited high enrichment scores (Figures 3c,d, S4b). The high protein content of L‐s CTL EVs compared with H‐s CTL EVs may be due in part to the ECM protein coating by interaction with integrins. In fact, coated membrane, anchored membrane, and membrane raft were represented in a GO cellular component analysis (*p* < 0.01) as L‐s CTL EV proteins compared with H‐s CTL EV proteins (Figure 3d).

**FIGURE 4:**
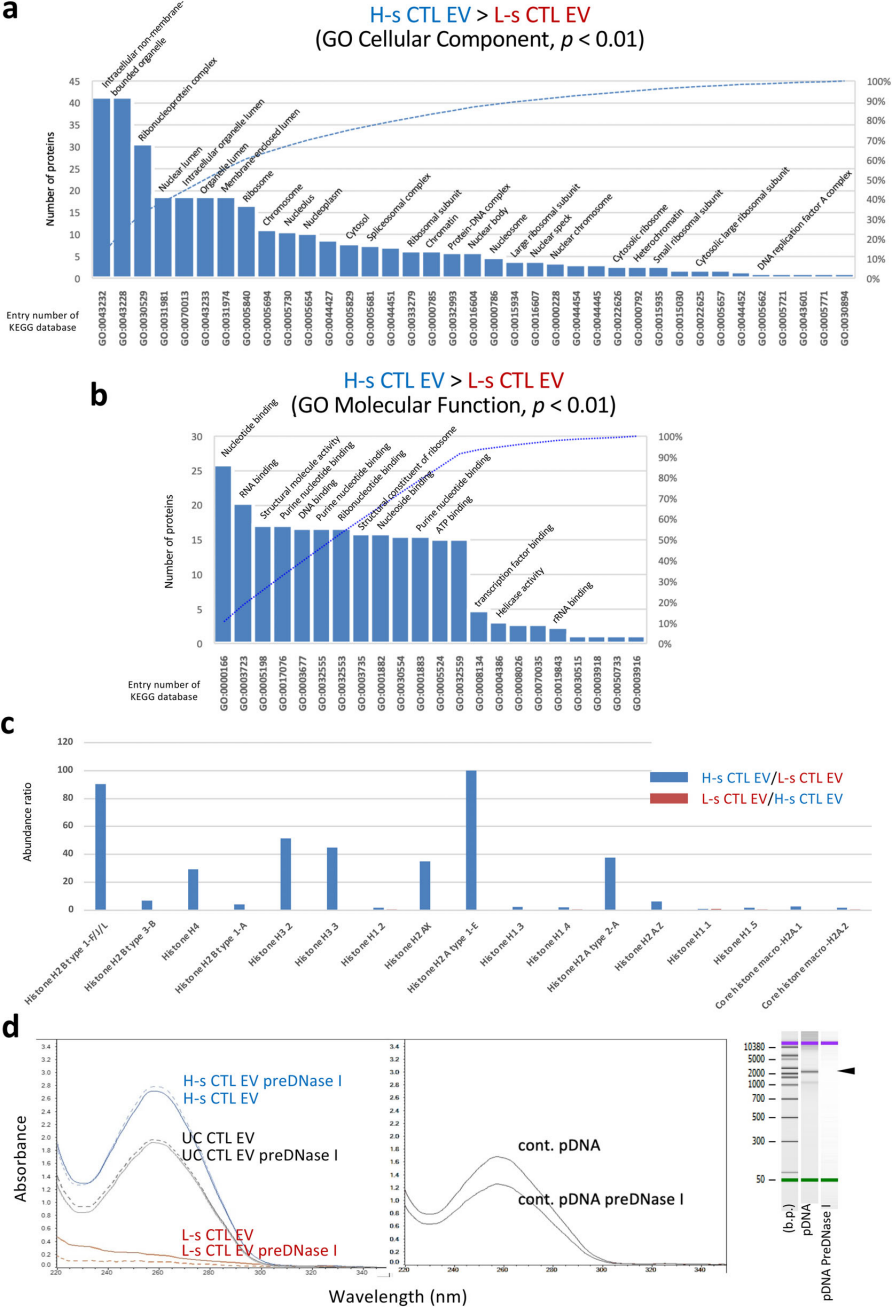
H‐s CTL EVs as a Nucleic Acid Cargo. (a and b) H‐s CTL EV‐dominant proteins (*p* < 0.01, Fisher's extract test), compared with L‐s CTL EV proteins were analyzed using GO cellular component and GO molecular function. (c) Abundance ratio of the histone proteins was calculated in cases of H‐s CTL EVs/L‐s CTL EVs (blue) and L‐s CTL EVs/H‐s CTL EVs (red) using normalized raw data. (d) DNA was extracted from 5 × 10^9^ particles of L‐s, H‐s, or UC CTL EVs pretreated with/without DNase I, and the UV absorbance was measured (*left*). To confirm the DNase I activity, 2.2 kbp plasmid DNA treated with/without DNase I at the same concentration with the EV treatment was extracted by DNA extraction kit, and the UV absorbance was measured (*middle*). The degradation of plasmid DNA was visualized through electrophoresis using a bioanalyzer (*right*). Analyzes of the pathways, GO cellular component, and GO molecular function were conducted using KEGG database in DAVID Bioinformatics Resources 6.8. Statistical significance was set at *p* < 0.05, and only *p *< 0.01 was shown.

H‐s CTL EVs were closely associated with ribosomes, chromosomes, RNA‐binding proteins, and DNA‐binding proteins based on the GO cellular component, GO molecular function, and pathway analyses (*p* < 0.01) (Figures 4a,b, S4c), which clearly indicated their role as a nucleic acid cargo. Core histone H2A, H2B, H3, and H4 proteins were found only in H‐s CTL EVs, but not in L‐s CTL EVs (Figure 4c), predicting the presence of DNA. DNA was extracted from L‐s, H‐s, and UC CTL EVs (5 × 10^9^ particles) with/without pre‐treatment with DNase I (under a degradation of plasmid (p) DNA, middle and right sides in Figure 4d), and the UV absorbance of each sample was measured. As expected, DNA was detected only in H‐s and UC CTL EVs, but not in L‐s CTL EVs (Figure 4d, left). UC CTL EVs exhibited less DNA content than H‐s CTL EVs, indicating a mixture of L‐s and H‐s CTL EVs. In addition, the DNA content of H‐s and UC CTL EVs was not changed through a pre‐treatment with DNase I, indicating that DNA is not bound outside the H‐s CTL EV membrane. The presence of DNA (Vagner et al., 2018), housekeeping molecules, cytoskeleton molecules, H‐2 class II molecules (Kowal et al., 2016; Ramachandra et al., 2010), and ARF6 (Pezzicoli et al., 2020) considers that H‐s CTL EVs are an MV‐like particle, because these substances are crucial for the evaluation of large EVs. Proteome analysis extracted 26 molecules that are present almost exclusively from the data of abundance ratio in H‐s CTL EVs (Figure S3d). In the future, these molecules may attract attention as MV‐specific markers.

It has been widely thought that EXOs have a lipid raft‐like membrane structure (de Gassart et al., 2003). To confirm this, the membrane lipid composition of L‐s and H‐s CTL EVs was examined through a lipidomic analysis using LC‐MS/MS. Among L‐s CTL EVs, H‐s CTL EVs, and UC CTL EVs, phosphatidylcholine (PC), phosphatidylethanolamine (PE), phosphatidylinositol (PI), and PS were predominantly high in phospholipids, and sphingomyelin (SM), ganglioside GA1 (asialo‐GM1), ganglioside GA2 (asialo‐GM2), and G1 ceramide (Cer) were high in sphingolipids, whereas the proportion of PC seemed to be lower than that in the previous report on EV lipid composition (Zhang et al., 2018) (Figures S5a,c). In SM, PC, PE, and PS, there was no unusual structure in the length of the short‐ and long‐chain fatty acids, or the position of the double bond (Figures S5b,d). Contrary to expectations, the ratio of sphingolipids to phospholipids was slightly greater in L‐s CTL EVs than in H‐s CTL EVs (Figure S5e). Because it has been reported that the phospholipids of the activated T cell membrane are present at approximately 10‐times greater levels more than sphingolipids (Zech et al., 2009), EV membranes accounting for approximately 30% of sphingolipids must be microdomain‐like membranes regardless of the EV type. These results indicate for the first time that the DEAE anion‐exchange method can separate bioactive EXOs from other MV‐like EVs as a nucleic acid cargo.

**FIGURE 5:**
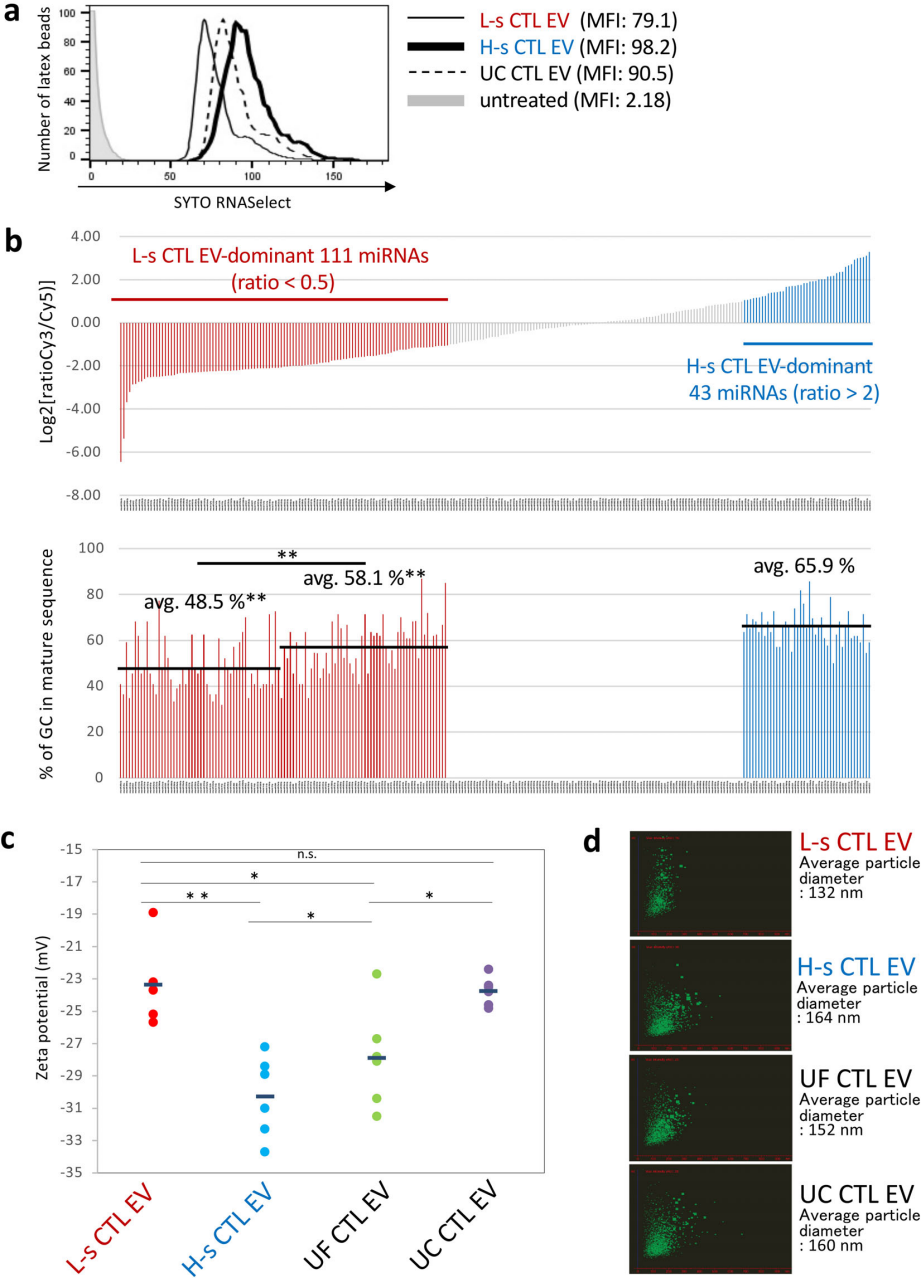
Differences in miRNA Composition and Zeta Potential Value between L‐s and H‐s CTL EVs. (a) To examine the amount of total RNAs, SYTO RNASelect‐stained L‐s, H‐s, and UC CTL EVs were adsorbed on latex beads and analyzed using a flow cytometer. (b) *Upper*. In global normalized data of L‐s and H‐s CTL EV mRNAs, miRNAs of less than 100 were deleted, and miRNAs having a variation ratio of less than 0.5 as L‐s CTL EV‐dominant 111 miRNAs and of more than 2 as H‐s CTL EV‐dominant 43 miRNAs were extracted and indicated in order from the lowest Log2 [ratio]. *Lower*. The GC % of L‐s CTL EV‐dominant 111 miRNAs and H‐s CTL EV‐dominant 43 miRNAs were calculated from the sequence of mature form referenced in miRBase (http://www.mirbase.org), and indicated in same order of miRNAs with upper figure. Statistical significances of GC % of among the first half of 55 and the last half of 56 L‐s CTL EV‐dominant miRNAs, and H‐s CTL EV‐dominant 43 miRNAs were determined by Student's *t*‐test. (**p < 0.01). (c) L‐s, H‐s, UF, and UC CTL EVs were prepared at 1 × 10^9^ particles/ml in a phosphate buffer with 0.015 M NaCl, and zeta potential values (mV) were examined six times. The negative zeta potential values of L‐s CTL EVs were significantly lower than those of H‐s CTL EVs or UF CTL EVs, and were similar to those of UC CTL EVs (*n* = 6; **p < 0.01, *p < 0.05, n.s., not significant, Student's *t‐*test). (d) Particle sizes of L‐s, H‐s, UF, and UC CTL EVs in a PBS suspension before the measurement of zeta potential value were examined through a nano‐tracking analysis. The particle size was measured three times, and the average particle size was determined. The data is representative of three nano‐tracking analysis images.

### 3.4 Differences in miRNA distribution, zeta potential value, target cells, and surface glycosylation between L‐s and H‐s CTL EVs

We next examined the differences in the properties of physiologically relevant between L‐s and H‐s CTL EVs. Using the SYTO RNASelect to determine the total EV RNAs, the amount of RNAs were in order of H‐s, UC, and L‐s CTL EVs with slight differences (Figure 5a). The miRNA analysis revealed 111 and 43 miRNAs as L‐s and H‐s CTL EV‐dominant miRNAs, respectively (Figure 5b, upper). Surprisingly, it was clarified by the calculation of GC % from the sequence of mature form of miRNAs that the GC % of L‐s CTL EV miRNAs was significantly smaller than that of H‐s CTL EV miRNAs (avg. 65.9%) in order of the strength of dominancy (avg. 48.5% and 58.1%) (Figure 5b, lower). In addition, L‐s CTL EVs were rich in Let‐7s and miRNAs with known functions, whereas H‐s CTL EVs mainly contained the four‐digit number miRNAs with unknown function (Tables S2, S3). Under these circumstances, MV‐like H‐s CTL EVs may participate in the disposal of dangerous DNA and GC‐rich miRNAs from parent cells.

Since 80% of UF CTL EVs retain their particle shape in water even after 24 h at 4℃, similar to a previous report from other group (Nguyen et al., 2016) (Figure S6), we measured the zeta potential values of L‐s, H‐s, UF, and UC CTL EVs in a phosphate buffer containing a 1/10 saline concentration (0.015 M NaCl). As indicated in Figure 5c, the negative zeta potential values were significantly larger in order of H‐s CTL EVs, UF CTL EVs, and L‐s CTL EVs. Although the negative zeta potential of UC CTL EVs showed weak values similar to those of L‐s CTL EVs, they appeared to be significantly different in morphology. L‐s CTL EVs were well dispersed, whereas large particles were observed in UC CTL EVs resemble with H‐s CTL EVs maybe reflecting particle aggregation (Figure 5d). This means that the electrostatic repulsive force is greater in the H‐s CTL EVs, but the dispersibility is superior in the L‐s CTL EVs.

**FIGURE 6:**
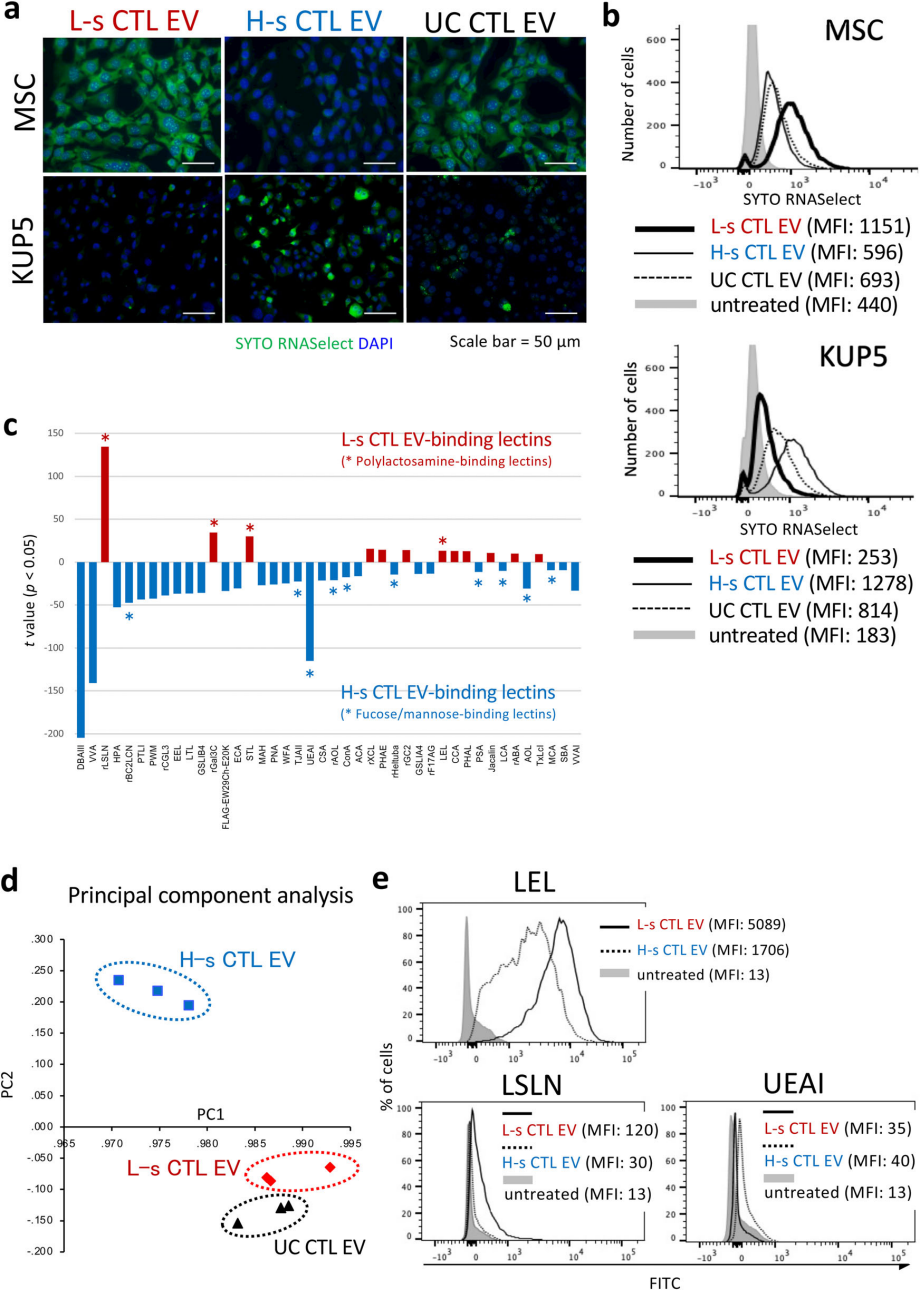
Differences in Target Cell Specificity and Membrane Glycan Structure between L‐s and H‐s CTL EVs. (a, b) L‐s, H‐s, and UC CTL EVs stained with SYTO RNASelect were added to the culture of MSCs or Kupffer cells, and incubated for 4 or 2 h, respectively. The green fluorescence engulfed by MSCs or Kupffer cells was observed with a fluorescence microscope (a) and flow cytometer (b). (c) A high‐density lectin microarray with immobilized 96 lectins was used to predict the membrane glycan structure. Lectin microarray data were mean‐normalized, and statistical significance was calculated using student's *t‐*test (See Figure S8). Forty‐two lectins with significantly different signals (*, *p *< 0.01) between L‐s CTL EVs and H‐s CTL EVs were arranged in ascending order of *p*‐values from the left, and their *t*‐values were displayed. (d) Principal component analysis of mean‐normalized data of lectin microarray was conducted among the L‐s, H‐s, and UC CTL EVs in three independent samples by using SPSS software. (e) L‐s and H‐s CTL EVs bound with latex beads were stained with FITC‐conjugated LEL, LSLN, and UEAI, and analyzed by flow cytometry.

In our recent study, it was demonstrated that i.t. administered UC CTL EVs are preferentially taken up by tumoral mesenchymal cells (Seo et al., 2018). Another group has reported that UC‐prepared tumour EVs are rapidly trapped and removed in the liver when administered intravenously (Matsumoto et al., 2017). Both reports predict that the EVs have different target directivities depending on the particle type. SYTO RNASelect‐stained L‐s, H‐s, and UC CTL EVs were treated in the cultures of mesenchymal stem cells (MSCs) and Kupffer cells (KUP5, immortalized murine Kupffer cell line; Kitani et al., 2014). As we thought, L‐s CTL EVs were preferentially engulfed by MSCs, but not H‐s CTL EVs. By contrast, phagocytosis by Kupffer cells was observed only in a H‐s CTL EV‐treated case (Figures 6a,b, S7). UC CTL EVs had an intermediate affinity for both MSCs and Kupffer cells in early phase.

Finally, we investigated the differences in surface glycosylation patterns between L‐s and H‐s CTL EVs through a lectin microarray analysis, and extracted statistically significant 42 lectins as L‐s CTL EV‐ and L‐s CTL EV‐binding lectins from 96 lectins (Figures 6c, S8). A multivariate analysis (principal component analysis) showed significant differences in glycosylation patterns between L‐s and H‐s CTL EVs rather than between L‐s and UC CTL EVs (Figure 6d). In addition, L‐s CTL EVs had an affinity for polylactosamine‐binding lectins such as *Laetiporus sulphureus* lectin (LSL‐N), galectin 3C (Gal3C), *Solanum tuberosum* lectin (STL), and *Lycopersicon esculentum* lectin (LEL) (Figures 6c,e). By contrast, H‐s CTL EVs bound with fucose‐ and mannose‐recognizing lectins such as *Burkholderia cenocepacia* lectin (BC2LCN), *Trichosanthes japonica* agglutinin‐II (TJA‐II), and *Ulex europaeus* agglutinin‐I (UEA‐I) (Figures 6c,e), which must be associated with a recognition by mannose‐ and fucose‐receptor‐expressing macrophages, including Kupffer cells. In fact, mannose and fucose receptors (CD206, CD207, and CD209a) were dominantly expressed in Kupffer cells (Figure S9).

## 4 DISCUSSION

Through the anion‐exchange separation of EVs in the culture supernatant, we clarified that EXOs can be distinguished from other cargos for nucleic acids based on the difference in the negative surface charge. Our results indicate the possibility that late endosome‐derived EXOs have less PS on the outer leaflet than the cargos for nucleotide acids, probably derived from the plasma membrane. Some other reports have also shown that EVs with tetraspanin molecules exhibit weaker annexin V‐ and lactadherin‐binding capacities than particles without them (Heijnen et al., 1999; Jeppesen et al., 2019; Matsumura et al., 2019). The Ca^2+^‐dependent activation of scramblase as TMEM16F is essential for MV formation, and MVs generated by budding from the plasma membrane express large amounts of PS in the outer leaflet of lipid bilayer membrane (Fujii et al., 2015; Nagata et al., 2016). Although knowledge regarding the mechanism of PS retention in the inner leaflet of EXO membrane is lacking, scramblase may not be present in the endosome membrane, and even if they are present, there may be a mechanism that is not activated, such as the acidic condition (pH range of 6.5 to 5, due to the activity of ATP‐proton pumps; Diering & Numata, 2014) of the endosomal system. Recently, it has been reported that scramblase on the plasma membrane loses its function at low pH in the cytoplasm (Liang & Yang, 2021).

The repulsive force between two negative charges is significantly greater for H‐s CTL EVs than for L‐s CTL EVs, which reflects the result that H‐s CTL EVs exhibit higher negative zeta potential values, and are eluted at higher salt concentrations than L‐s CTL‐EVs. A lipidome analysis showed no dramatic difference in the PS ratio among L‐s, H‐s, and UC CTL EVs, suggesting another possibility that PS in the outer leaflet of the EV membranes may not be crucial for separating EXOs from other particles with anion‐exchange method. Since LSL‐N lectin, which recognizes the galactose of polylactosamine without negative charged sialic acid, dominantly binds with L‐s CTL EVs, L‐s CTL EVs may have a weaker negative charge than H‐s CTL EVs by their low content of surface sialic acids.

Notably, L‐s CTL EVs express various integrins, in addition to the high expression levels of EXO markers including CD9 and Tsg101. One speculation is that EXOs are covered via integrins with ECM proteins such as thrombospondins, which are known as T cell‐producing ECM proteins (Li et al., 2002). In a proteome analysis, thrombospondins were detected in L‐s CTL EVs at the same ratio of EV markers (Figure S10a). Importantly, it has been reported that the negative zeta potential value is reduced without increasing the cohesion by an offset of electrostatic repulsion between two negative charges of EVs with CD63‐specific mAb, indicating a decrease in cohesiveness owing to the spread of the interparticle distance (Akagi et al., 2015). Consistent with this phenomenon, coating with ECM proteins may be necessary for EXOs to circulate systemically without agglutination even if the negative zeta potential value is small. L‐s CTL EV wears certain ECM proteins via integrins, and UC CTL EV wears contaminant proteins derived from culture medium (Linares et al., 2015), which is considered to be the reason why the negative zeta potential values are equivalent in both EVs.

L‐s CTL EVs are easily taken up by mesenchymal cells, and H‐s CTL EVs are preferentially engulfed by Kupffer cells in vitro. Since L‐s and H‐s CTL EVs are respective recognized by polylactosamine‐specific lectins and fucose/mannose‐binding lectins, the interaction of the receptors on the target cells with glycans on EV surfaces may explain the difference in the target specificity. Otherwise, since polylactosamines of N‐glycans regulate the T cell activity (Togayachi et al., 2007), polylactosamine on L‐s CTL EVs may also participate in regulation of T cell activity. Galactose of polylactosamines plays a pivotal role in signaling for endocytosis by interacting with galectins on target cells (Johannes et al., 2018). Galectin‐1 and ‐3 are known to be expressed on the MSC surface, which is one of the characteristics of MSCs (Gieseke et al., 2010; Sioud et al., 2011), suggesting that the interaction between galectins and polylactosamines is important for the MSC specificity of L‐s CTL EVs. The receptors for fucose/mannose glycans such as CD206, CD207, and CD209a are known to be expressed in M2‐type macrophages and Kupffer cells (Kerrigan & Brown, 2009; Nielsen et al., 2018; Stahl & Gordon, 1982), and it is possible to explain the interaction of Kupffer cells with H‐s CTL EVs. The phagocytosis of MV‐like H‐s CTL EVs by M2 macrophages and Kupffer cells must be a clearance system of unnecessary and dangerous nucleic acids from the parent cells. However, our previous study shows no engulfment of CD206^+^ tumour‐associated macrophages by i.t. administration of UC CTL EVs (Seo et al., 2018), considering that tumour‐associated macrophages are in different stages of differentiation from conventional M2 macrophages and Kupffer cells, and do not participate in the elimination of MV‐like EVs as a nucleic acid cargo. It has been reported that DNA‐containing CD4^+^ T cell EVs are taken up by dendritic cells (DCs) during antigen‐specific interaction, and induce interferon‐regulated gene‐mediated antiviral responses through cytosolic DNA sensors (Torralba et al., 2018). H‐s CTL EVs may have an important biological significance in the acquisition of resistance to viral infections, but not tumours, when they are engulfed by DCs. The differences in the novel properties between L‐s CTL EVs and H‐s CTL EVs are summarized in Figure S10b.

Establishing a novel method for preparation of large amounts of highly purified EVs has become the central issue for EV drug discovery in recent years. Although EVs prepared by the density gradient and affinity methods are excellent for isolation, a large‐scale preparation is difficult, and the reliability of biological activity remains ambiguous. EVs obtained by UC is dirty and cannot be evaluated for reliable biological activity. In this study, we developed the effective EV preparation method that enable a large quantity at high‐purity without decreasing bioactivity. In addition, this method can also clearly distinguish between bioactive EXOs and other MV‐like EVs as a nucleic acid cargo (Figure S11). Therefore, the anion‐exchange method, which overcomes all issues of the current EV preparation method, must be the gold standard for the therapeutic EV preparation in the future.

## AUTHOR CONTRIBUTIONS

Conceptualization, N.S.; Methodology, N.S. and T.K.; Investigation, N.S. (Biological studies in vitro and in vivo, Acquisition, analysis, and deposition of comprehensive data, DNA extraction, Flowcytometric analysis of cells, and EV preparation by UC, UF, and ion exchange methods), J.N. (NTA analysis, Western blotting, EV preparation by UF and ion exchange method, Measurement of UV absorbance, and Flowcytometric analysis of EVs), T.K. (EV preparation by ion exchange method), H.T., (Lectin microarray analysis) and A.S. (Measurement of zeta potential); Writing, Original Draft, N.S.; Review and editing of MS, T.K., T.I., K.F., J.H., K.A., and H.S.; Funding Acquisition, N.S. and K.A., and Supervision, N.S.

## CONFLICT OF INTERESTS

The authors report no conflict of interest.

## Supporting information

Supplementary information
